# Tumor kinetics prior to treatment correlate with response to checkpoint inhibitors

**DOI:** 10.1186/2051-1426-3-S2-P252

**Published:** 2015-11-04

**Authors:** Alexandra P Cadena, James Welsh, Chad Tang, Uma Raju, David Hong, Aung Naing, Myrna Godoy, Pamela K Allen, Jonathan Schoenhals, Maria Angelica Cortez

**Affiliations:** 1 The University of Texas MD Anderson Cancer Center, Houston, TX, USA

## 

Immunotherapy has become one of the most profound advances in the treatment of solid tumors. Unlike the prior targeted therapies such as kinase inhibitors, there are no clear mutations or definitive biomarkers of which patients are likely to respond. It is well documented that many patients with PDL1 negative tumors can still have profound responses to PD1 inhibitors. As such there is a critical need for predictors of response to these new therapeutics. We hypothesize that patient tumor growth kinetics correlate with endogenous ability to suppress tumor growth and will predict overall benefit from checkpoint inhibitors.

To establish this correlation, a retrospective study was done that reviewed chest CTs from five patients with an iRECIST of partial response and five non-responders with an iRECIST of progressive disease. The ten patients selected completed an ipilimumab treatment regimen and had sufficient diagnostic imaging that dated back at least 18 months prior to the start of their immunotherapy treatment. The growth curves (pre-treatment) of three lesions were obtained for each patient and then averaged. The tumor growth kinetics were followed over four time points (+/- 2 months) starting with 18 months prior to immunotherapy treatment and ending at 2 months prior to treatment. Slopes of the obtained curves were done on a linear scale.

**Figure 1:**
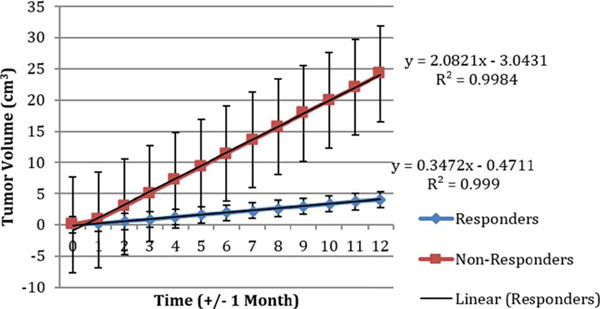
Tumor kinetics of responders vs. non-responders as a function of time.

A total of 10 patients were evaluated. The median age was 54, the average number of prior treatments was 3, and the tumor types varied, including melanoma, non-small cell, colorectal, and anaplastic thyroid. We found that the linear slope of the growth kinetics for the responder patients was 0.3427 and the slope of the tumor growth kinetics for the non-responder patients was 2.0821 (Figure 1). The p-value according to the student's t-test between the two slopes was 0.15.

There is great enthusiasm for the integration of checkpoint inhibitors in solid tumors. Unfortunately the majority of patients do not respond. Presently there are no approved biomarkers to predict which patients are most likely to benefit from immunotherapeutics. We evaluated tumor growth kinetics prior to the initiation of treatment and found that this is a simple way to obtain prognostic information about the potential benefit from immunotherapies. Tumor growth kinetics likely reflect the biology of a tumor-host interaction; further work needs to be done to validate these results and potential biology in retrospective and prospective studies.

